# *Staphylococcus aureus* type I signal peptidase: essential or not essential, that’s the question

**DOI:** 10.15698/mic2017.04.566

**Published:** 2017-03-17

**Authors:** Wouter L. Hazenbos, Elizabeth Skippington, Man-Wah Tan

**Affiliations:** 1 Department of Infectious Diseases, Genentech Inc., 1 DNA Way, South San Francisco, CA 94080, USA.; 2 Department of Bioinformatics and Computational Biology, Genentech Inc., 1 DNA Way, South San Francisco, CA 94080, USA.

**Keywords:** bacterial secretion, signal peptidase, bacterial resistance

## Abstract

Secretion of proteins into the extracellular environment is crucial for the normal physiology and virulence of pathogenic bacteria. Type I signal peptidase (SPase I) mediates the final step of bacterial secretion, by cleaving proteins at their signal peptide once they are translocated by the Sec or twin-arginine (Tat) translocon. SPase I has long been thought to be essential for viability in multiple bacterial pathogens. Challenging this view, we and others have recently created *Staphylococcus aureus* bacteria lacking the SPase I SpsB that are viable and able to grow *in vitro* when over-expressing a native gene cassette encoding for a putative ABC transporter. This transporter apparently compensates for SpsB's essential function by mediating alternative cleavage of a subset of proteins at a site distinct from the SpsB-cleavage site, leading to SpsB-independent secretion. This alternative secretion system also drives the main mechanism of resistance to an arylomycin-derived SpsB inhibitor, by means of mutations in a putative transcriptional repressor (*cro/cI*) causing over-expression of the ABC transporter. These findings raise multiple interesting biological questions. Unraveling the mechanism of SpsB-independent secretion may provide an interesting twist to the paradigm of bacterial secretion.

*Staphylococcus aureus* is an important human pathogen that can cause life-threatening invasive infections, such as bacteremia, endocarditis, pneumonia, and osteomyelitis. Because of its nominal essentiality, Type I signal peptidase (SPase I) has been investigated as a potential antibacterial target. Several factors make *S. aureus* SpsB an attractive antibiotic target 1. First, the enzymatic domain, consisting of the serine-lysine dyad, is unique to prokaryotes and is druggable; inhibitors of the enzyme have been described previously in the literature 1. Second, the extracellular location of the enzymatic pocket makes this target relatively accessible as it obviates the requirement of the inhibitor to traverse the bacterial membrane. Recently, derivatives of the arylomycin family of SpsB inhibitors with enhanced potency against *S. aureus* have been generated 2,3.

**Figure 1:**
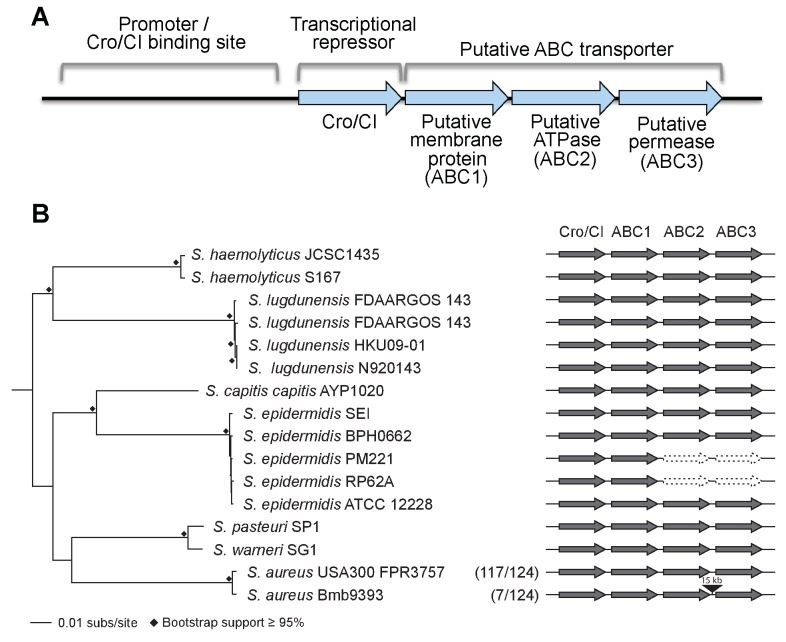
FIGURE 1: Genomic organization of the operon of the Cro/CI and ABC transporter operon in *Staphylococcus*. **(A)** Functional model for the operon in *S. aureus*. The *S. aureus cro/cI* gene is a homolog of the lambda phage transcriptional repressor *cro*, and shares an operon with three genes encoding a putative ABC transporter (abbreviated as ABC1, ABC2, and ABC3). *S. aureus* Cro/CI functions as a transcriptional regulator, suppressing transcription of all four operon genes. The putative binding site of the Cro/CI protein presumably lies just upstream of the *cro/cI* gene in its promoter area. Mutations in *cro/cI* lead to 40~100 fold over-expression of the putative ABC transporter, causing resistance to arylomycin derivatives. Genes and promoter area are not drawn to scale. For more details see reference 3. **(B)** Schematic showing the genomic organization of the *cro*/*cI* - ABC transporter operon in selected pathogenic *Staphylococcus* genomes. The intact operon is well conserved in these staphylococci. Exceptions are the lack of ABC2 and ABC3 encoding genes in 2 of 5 *S. epidermidis* genomes, and an ~15 kb insertion between ABC2 and ABC3 in 7 of 124 *S. aureus* genomes. The phyletic distribution of *cro*/*cI* and the ABC transporter genes was determined using BLAST+ 8. The maximum-likelihood (RAxML 9, GTRGAMMA) phylogeny was constructed on the basis of a concatenated core genome alignment generated using Mugsy 10. Nodes with ≥ 95% bootstrap support from 1,000 replicates are indicated by black diamonds. Present genes are dark gray, absent genes are white with a dotted outline, and the insertion is black. In parentheses are the numbers of *S. aureus *strains of the total examined that share the genomic organization of the representative strains shown.

A surprising observation from resistance studies performed by our group and others is that mutations in the locus encoding for the putative transcriptional regulator *cro/cI* confer resistance to arylomycin-derived SpsB inhibitors in *S. aureus* 3,4. The Cro/CI protein shares sequence similarity with lambda phage Cro, and has transcriptional repressor activity in *S. aureus* 3. Loss-of-function mutations in the *cro/cI* locus are associated with over-expression of the 3-gene locus immediately downstream of *cro/cI* which encodes a putative ABC transporter 3,5 (see Figure 1A). Over-expression of the ABC transporter is sufficient to confer resistance to SpsB inhibitors, whereas over-expression of the transporter with an inactivated ATPase domain is not 3. This suggests that this resistance mechanism is ATP-dependent and possibly mediated by active transport 3. Over-expression of the ABC transporter overcomes the *S. aureus* lethality caused by either pharmacologic inhibition or genetic ablation of SpsB, and is associated with secretion of alternatively cleaved proteins 3,5 (see Figure 2 for a model). Thus, over-expression of the putative ABC transporter is both necessary and sufficient to compensate for the essentiality of *S. aureus* SpsB *in vitro*. Together these findings introduce a new concept, which challenges the common belief that SpsB is absolutely essential for viability, and which invites a number of interesting questions.

**Figure 2:**
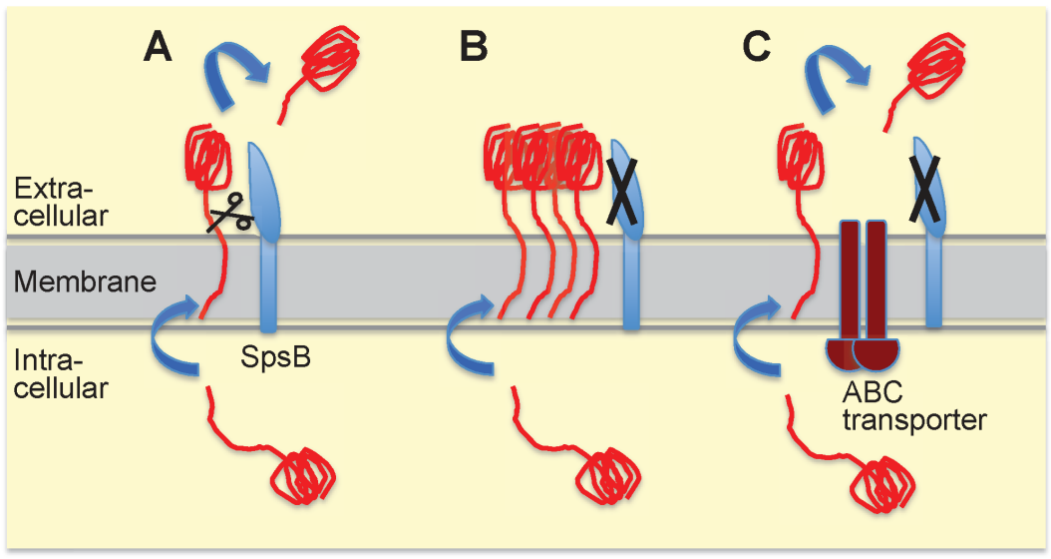
FIGURE 2: Model for alternative secretion compensating for the lack of type I signal peptidase SpsB activity in *S. aureus*. **(A)** The most common route for proteins destined for secretion involves translocation across the *S. aureus* cell membrane through the general secretory (Sec) translocon, and subsequent cleavage by SpsB of the signal (or leader) peptide allowing release from the membrane. SpsB also cleaves the N-terminal signal peptides of proteins secreted through the Tat protein export system. **(B)** When SpsB is inhibited, these proteins are not cleaved and hence not secreted, causing accumulation of unprocessed proteins, and eventually resulting in cell death. **(C)** Over-expression of the putative ABC transporter (as in *S. aureus* with mutations in the transcriptional repressor *cro/cI*) compensates for a lack of SpsB activity. This transporter mediates SpsB-independent cleavage of a subset of proteins at an alternative cleavage site, leading to their secretion. This alternative secretion pathway is able to restore viability under conditions of either pharmacological SpsB inhibition (in which case it constitutes the main resistance mechanism) or genetic disruption of SpsB expression. For more details see reference 3.

First, to what extent does the putative ABC transporter compensate for the absence of functional SpsB? Proteomic studies of the secretome of a wild-type *S. aureus* strain and an *S. aureus* strain that lacks SpsB while over-expressing the ABC transporter show that only a minor subset of proteins that are normally SpsB-cleavable is alternatively secreted 3,5. These proteins, which are secreted independently of SpsB, are cleaved at a site N-terminal to the canonical SpsB-cleavage site 3. Given that the ABC transporter does not encode any domain that resembles a protease, it is feasible that one of its substrates posesses the proteolytic activity responsible for this alternative cleavage.

The observation that the alternative cleavage occurs N-terminal of the SpsB cleavage site suggests the involvement of a membrane-localized protease. Although the strain lacking SpsB and over-expressing the ABC transporter appears to grow well *in vitro*, it is unable to establish infection in a mouse model 3. Pharmocological inhibition of SpsB has been shown to reduce secretion of several *S. aureus* virulence factors 6. Notably, when SpsB expression is genetically disrupted, over-expression of the ABC transporter is not able to sufficiently restore secretion of several specific virulence factors known to contribute to establishment of infection, including adhesion molecules and secreted proteases 3. Thus, the rather limited subset of proteins that are alternatively secreted through the ABC transporter in the absence of SpsB uncouples *in vitro* bacterial growth from *in vivo* infectivity. An interesting consideration that follows is that since *S. aureus* is a commensal of mammalian skin and nares 7, the strong conservation of SpsB may thus be a consequence of its essentiality for viability within these niches.

Second, by which mechanism does the alternative ABC transporter-mediated secretion pathway compensate for the essentiality of SpsB *in vitro*? To speculate on the answer, it would be relevant to understand the mechanism by which SpsB inhibition leads to bacterial death. Two main hypotheses, that are not mutually exclusive, can be proposed. First, in the absence of SpsB activity, cell death may be caused by accumulation of unprocessed proteins leading to disruption of membrane integrity. This hypothesis raises the possibility that the ABC transporter is directly or indirectly involved in removal of unprocessed proteins. It is possible that the transporter enables a hypothetical membrane-associated proteolytic enzyme to degrade these unprocessed membrane proteins. Second, under SpsB inhibitory conditions, cell death may occur because certain secreted proteins that are essential for viability cannot be secreted. In the context of this hyopothesis, it can be speculated that in the absence of SpsB, the ABC transporter enables secretion of an individual protein or a combination of proteins that is otherwise essential. Although such proteins have not been identified as yet, this possibility also cannot be excluded. Future experiments to elucidate the mechanism by which the ABC transporter compensates for the essentiality of SpsB should help address this question.

Third, what is the physiological role of the putative ABC transporter? Over-expression of the ABC transporter to overcome a loss of secretion may be a protective mechanism in a natural environment in which arylomycin-like antibacterials are present, and this could be relevant for niche competition. In this context, it is noteworthy that inactivation of the ABC transporter (in the absence of *cro/cI* mutations) renders *S. aureus* much more sensitive to treatment with an arylomycin-derived SpsB inhibitor in mice 3. This raises the possibility that the natural function of the ABC transporter represents a phenotypic resistance mechanism, perhaps through its over-expression in response to arylomycin-like antibacterials present in the environment. This would be consistent with the observation that treatment with an arylomycin derivative transiently upregulates transcription of a homolog of the ABC transporter 4. At present, it is unknown which transcriptional regulator may be involved in this phenotypic response, although *cro/cI* itself is a likely candidate. It is possible that *cro/cI* is part of an as yet to be defined signaling mechanism that senses the presence of arylomycin in a natural environment.

Fourth, is the Cro/CI - ABC transporter operon universally conserved in pathogenic *Staphylococcus* species? We find the operon is remarkably conserved in some but not all pathogenic staphylococci. *Cro*/*cI *and the three downstream genes encoding the ABC transporter proteins (in this paper referred to as ABC1, ABC2, and ABC3; Figure 1) are found as an intact collinear unit in 133 of 145 *Staphylococcus *genomes examined. However, the operon is disrupted in a subset of strains in at least two pathogenic staphylococcal species. First, in two *S. epidermidis* genomes, the genes encoding ABC2 and ABC3 are missing entirely (Figure 1B). The loss of these two genes likely reflects the lack of a functional ABC transporter, as each is essential for resistance of a* S. aureus* USA300 *cro*/*cI* mutant to SpsB inhibitors 3. Second, in 7 of 124 *S. aureus* genomes analyzed, the operon has been interrupted by the obvious insertion of an ~15 kb transposable element between the ABC2 and ABC3 encoding genes (Figure 1B). While the gene encoding ABC3 in these strains may no longer be under the control of the *cro/cI* promoter, it is unclear whether it can still be expressed and whether an active ABC transporter can be formed. Thus, the operon is not universally intact in all *Staphylococcus *genomes; further experimental studies are needed to determine whether insertion of the transposable element in the *cro*/*cI* - ABC transporter operon inactivates SpsB-independent secretion.

In conclusion, the discovery of a potential new secretion system in *S. aureus* that is able to bypass the nominal essentiality of SpsB raises a number of interesting questions. Determining the molecular mechanism of this alternative secretion pathway has the potential to provide new insights into the basic biology of bacterial secretion and to aid design of new antibacterial therapies.

## References

[1] Rao S, De Waelheyns E, Economou A, Anné J (2014). Antibiotic targeting of the bacterial secretory
pathway.. Biochimica et Biophysica Acta.

[2] Therien AG, Huber JL, Wilson KE, Beaulieu P, Caron A, Claveau D, Deschamps K, Donald RG, Galgoci AM, Gallant M, Gu X, Kevin NJ, Lafleur J, Leavitt PS, Lebeau-Jacob C, Lee SS, Lin MM, Michels AA, Ogawa AM, Painter RE, Parish CA, Park YW, Benton-Perdomo L, Petcu M, Phillips JW, Powles MA, Skorey KI, Tam J, Tan CM, Young K, Wong S, Waddell ST, Miesel L (2012). Broadening the spectrum of β-lactam antibiotics through
inhibition of signal peptidase Type I.. Antimicrobial Agents and Chemotherapy.

[3] Morisaki JH, Smith PA, Date SV, Kajihara KK, Truong CL, Modrusan Z, Yan D, Kang J, Xu M, Shah IM, Mintzer R, Kofoed EM, Cheung TK, Arnott D, Koehler MFT, Heise CE, Brown EJ, Tan MW, Hazenbos WLW (2016). A putative bacterial ABC transporter circumvents the essentiality
of signal peptidase.. mBio.

[4] Craney A, Romesberg FE (2015). A putative Cro-like repressor contributes to arylomycin
resistance in Staphylococcus aureus.. Antimicrobial Agents and Chemotherapy.

[5] Craney A, Dix MM, Adhikary R, Cravatt BF, Romesberg FE (2015). An alternative terminal step of the general secretory pathway in
Staphylococcus aureus.. mBio.

[6] Schallenberger MA, Niessen S, Shao C, Fowler BJ, Romesberg FE (2012). Type I signal peptidase and protein secretion in Staphylococcus
aureus.. Journal of Bacteriology.

[7] Wertheim HF, Melles DC, Vos MC, Van Leeuwen W, Van Belkum A, Verbrugh HA, Nouwen JL (2005). The role of nasal carriage in Staphylococcus aureus
infection.. Lancet.

[8] Camacho  C, Coulouris G, Avagyan V, Ma  N, Papadopoulos J, Bealer K, Madden TL (2009). BLAST+: architecture and applications.. BMC Bioinformatics.

[9] Stamatakis A (2006). RAxML-VI-HPC: maximum likelihood-based phylogenetic analyses with
thousands of taxa and mixed models.. Bioinformatics.

[10] Angiuoli SV, Salzberg SL (2011). Mugsy: fast multiple alignment of closely related whole
genomes.. Bioinformatics.

